# Laudanum Poisoning Successfully Treated by Belladonna

**Published:** 1872-12

**Authors:** 


Laudanum Poisoning Successfully Treated by Belladonna. Dr. Thomas Thatcher Graves, of Lynn, Mass., reports a case of poisoning by opium, says the *Boston Medical and Surgical Journal,* in which the patient, a male adult, had taken two ounces of Laudanum. Dr. Graves saw the patient two hours after he had taken the narcotic. The proof was positive that he had taken two full ounces of the drug, and that he had not vomited. He found the pupils greatly contracted; totally blind; not able to speak; radial pulse not perceptible; respiration slow, but number not noted; hands and feet cold and clammy. The patient was not insensible to sound. There was great muscular tremor, and at the time of his examination it did not seem possible for him to live fifteen minutes. He says: "I decided to administer tincture of belladonna. Carefully closing the epiglottis with my fingers, I poured one drachm of Squibbs' tincture of belladonna into the patient's mouth. In a very few moments he seemed so much revived that he swallowed another tea-spoonful of the same."

The Doctor states that, soon after the administration of the belladonna he became bathed in the most profuse perspiration; the pupils began to dilate, and he could speak—and expressed surprise—remembered taking the laudanum.

In fifteen or twenty minutes after the first dose, he gave him twenty drops of Thayer's fluid extract of belladonna, and afterwards one drop every five minutes for an hour. At the expiration of one hour the symptoms had so far subsided that no one would have regarded the case as one of opium poisoning.

Many of the toxical symptoms of belladonna were manifest for some hours after the administration of the remedy. The patient did well.

